# Machine learning-based microarray analyses indicate low-expression genes might collectively influence PAH disease

**DOI:** 10.1371/journal.pcbi.1007264

**Published:** 2019-08-12

**Authors:** Song Cui, Qiang Wu, James West, Jiangping Bai

**Affiliations:** 1 College of Agronomy, Gansu Agricultural University, Lanzhou, Gansu, China; 2 School of Agriculture, Middle Tennessee State University, Murfreesboro, Tennessee, United States of America; 3 Department of Mathematics, Middle Tennessee State University, Murfreesboro, Tennessee, United States of America; 4 Department of Medicine, Pulmonary Vascular Research Institute, Vanderbilt University Medical Center, Nashville, Tennessee, United States of America; Ottawa University, CANADA

## Abstract

Accurately predicting and testing the types of Pulmonary arterial hypertension (PAH) of each patient using cost-effective microarray-based expression data and machine learning algorithms could greatly help either identifying the most targeting medicine or adopting other therapeutic measures that could correct/restore defective genetic signaling at the early stage. Furthermore, the prediction model construction processes can also help identifying highly informative genes controlling PAH, leading to enhanced understanding of the disease etiology and molecular pathways. In this study, we used several different gene filtering methods based on microarray expression data obtained from a high-quality patient PAH dataset. Following that, we proposed a novel feature selection and refinement algorithm in conjunction with well-known machine learning methods to identify a small set of highly informative genes. Results indicated that clusters of small-expression genes could be extremely informative at predicting and differentiating different forms of PAH. Additionally, our proposed novel feature refinement algorithm could lead to significant enhancement in model performance. To summarize, integrated with state-of-the-art machine learning and novel feature refining algorithms, the most accurate models could provide near-perfect classification accuracies using very few (close to ten) low-expression genes.

## Author summary

Pulmonary arterial hypertension (PAH) is a serious and progressive disease, with only a roughly 50% of 5-year survival rate even with best available therapies. Accurately detecting/differentiating different forms of PAH and developing drugs that could directly target at genes involved in PAH pathogenesis are essential. We proposed a computational approach using low-cost microarray data collected from a clinical trial and had accurately predicted each PAH group. In particular, we considered the fact that there might exist some low-expression genes that were usually discarded by researchers but might function collectively and significantly controlling the disease in each case. Therefore, we had developed different filtering algorithms that intentionally selected those low-expression genes for constructing prediction model. Using a few highly informative low-expression genes that had never been extensively investigated before, our systematic approach had produced models that could offer prefect accuracy in predicting PAH. Additionally, our analysis also found that the composition of gene factors controlling the PAH etiology under each form are quite different from each other.

## Introduction

Pulmonary arterial hypertension (PAH) is a fatal and progressive disease characterized by increasing pulmonary vascular resistance leading to heart failure and death [1–4]. A significant amount of research has been conducted previously, which greatly enhanced understanding of the molecular mechanisms and etiology involved in PAH. However, the underlying interactive effects of different genetic mutations and fundamental mechanisms of vascular dysfunction remains unclear [4]. For example, mutation in bone morphogenetic protein (BMP) receptor type II (BMPR2) was found to be significantly correlated with the development of both heritable (HPAH) and the idiopathic form of PAH (IPAH) [5]. In another study, a known BMP signaling regulator, transcription factor MSX1, was found to be strongly correlated with IPAH cases [1]. Additionally, genes expression signatures from IPAH patients were either tightly clustered with HPAH group or in an isolated cluster [4]. These findings suggest that different forms of PAH might share the majority of the molecular origins/signaling pathways but there might exist some distinct factors modulating the primary genetic expression in each case [1]. Furthermore, the majority of the PAH cases in human beings were found to be unassociated with BMPR2 mutation [1], and some other factors have been identified to be partially contributing to IPAH [6,7]. We attributed the difficulty in fully unraveling the genetic factors causing PAH largely to the lack of high-quality patient data in conjunction with advanced data processing algorithms, limited comprehension of the genetic etiology, and overlook of some of the important low-expression genes that might interactively affect PAH as a whole.

Microarray-based gene sequencing provides a fast and cost-effective screening technology, and has been used in many PAH studies to identify important signaling pathways that could impact PAH pathogenesis [3,8,9]. Particularly, more than 25 microarray studies have been conducted on human PAH in the past ranging from single-gene expression to more complex pathway analyses, providing large quantity of data pertaining to PAH pathogenesis. Following common data processing protocols, the majority of these microarray data analysis routines involve background noise reduction and normalization. Additionally, the differentially expressed genes are typically ranked by their logarithmically transformed fold change values or by moderated t-statistic values. The low-expression genes [e.g. log-transformed probe intensity values < 2^7^ or 2^8^, or the inter-quartile range (IQR) detected threshold values] are typically considered unreliable and treated as ‘noises,’ which are usually removed from the dataset manually [10–12] or by applying the IQR filtering algorithm provided by software packages such as the Bioconductor [13, 14]. However, we expect that these low-expression genes might have significant interactive effects in PAH etiology and might become very informative when functioning collectively. We hypothesized that using advanced data-science algorithms in conjunction with high-quality clinical research data would reveal significant coexisting controlling factors and interactive genes (including those with low-expression values) that could impact PAH pathogenesis.

The primary goal of this study was to identify a small group of genes (including low-expression genes) and construct classification models that could accurately predict each patient’s PAH status. To accomplish this, we ranked and selected genes according to their contribution towards the model construction processes. Particularly, our research group had formulated a novel recursive feature elimination algorithm integrated with conventional machine learning data analysis paradigm. Three popular supervised machine learning algorithms were selected for the modeling processes, including Linear Discriminant Analysis (LDA), Support Vector Machine (SVM), and Artificial Neural Networks (ANN). All three algorithms rely on labelled training data to find a linear combination of features (e.g. LDA), a maximized margin of a decision boundary (e.g. SVM), or an optimized neuron-edge network structure (e.g. ANN) that could best separate data belonging to different groups. Additionally, this project investigated the possibility of constructing highly accurate prediction models in determining different forms of PAH, which, if worked, could provide great potential for future clinical usage and commercialization. The main dataset was obtained as Affymetrix array-based gene expression data from anticoagulated whole blood samples collected from 86 patients, which includes the healthy control group (22 patients), the IPAH group (20 patients, BMPR2 mutation excluded), the HPAH (17 patients) and the BMPR2 mutation carriers that have no clinical signs of disease (27 Unaffected Mutation Carriers, UMC). Our classification models indicated excellent performance in distinguishing each individual patient group (Control *vs*. IPAH *vs*. HPAH *vs*. UMC); and separating the control group *vs*. the combination of HPAH and IPAH, as well as the HPAH group *vs*. UMC. Additionally, different gene filtering criteria were used to test the assumption that there might exist clusters of important low-expression genes collectively controlling the forms of PAH in human beings.

## Results

**Fig 1:**
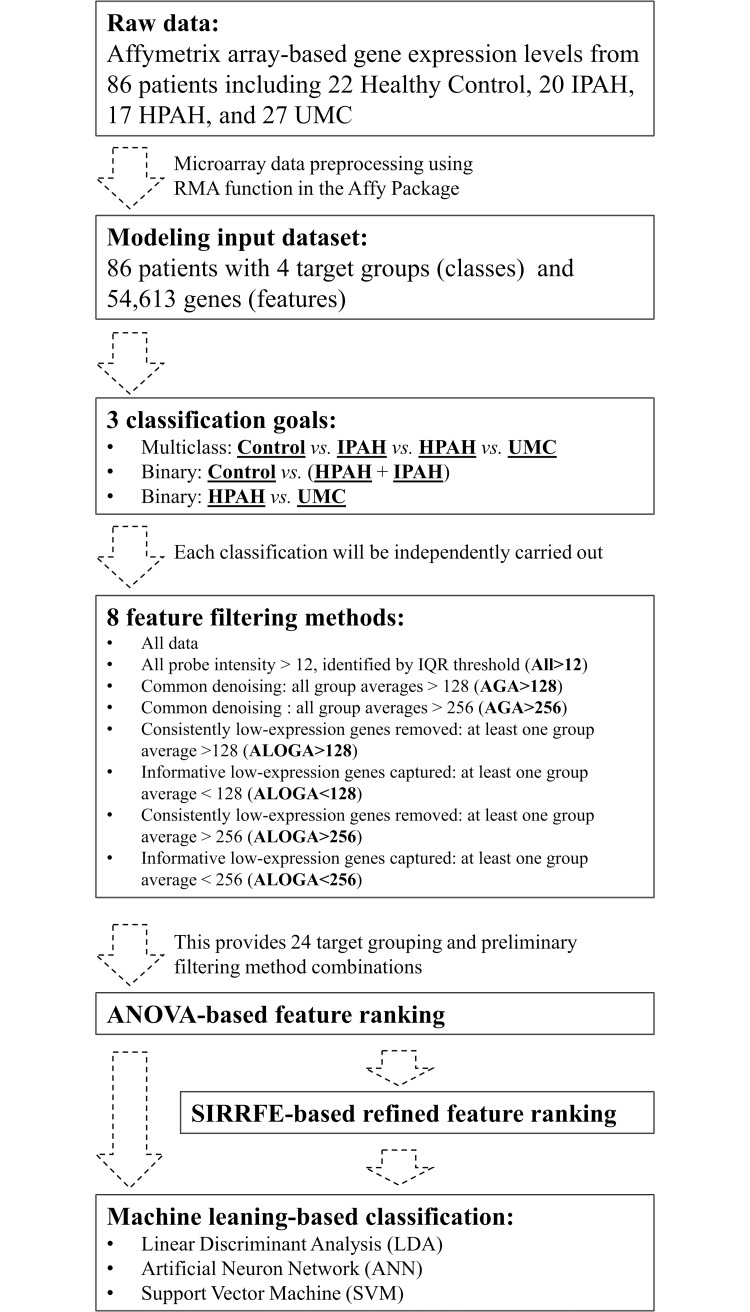
The overall data analysis design and logic flow. The first step was performed using the RMA function in the Affy Package. The rest of the workflow was implemented using the Matlab Programming Language version R2017a (The MathWorks, Inc., Natick, Massachusetts).

**Fig 2:**
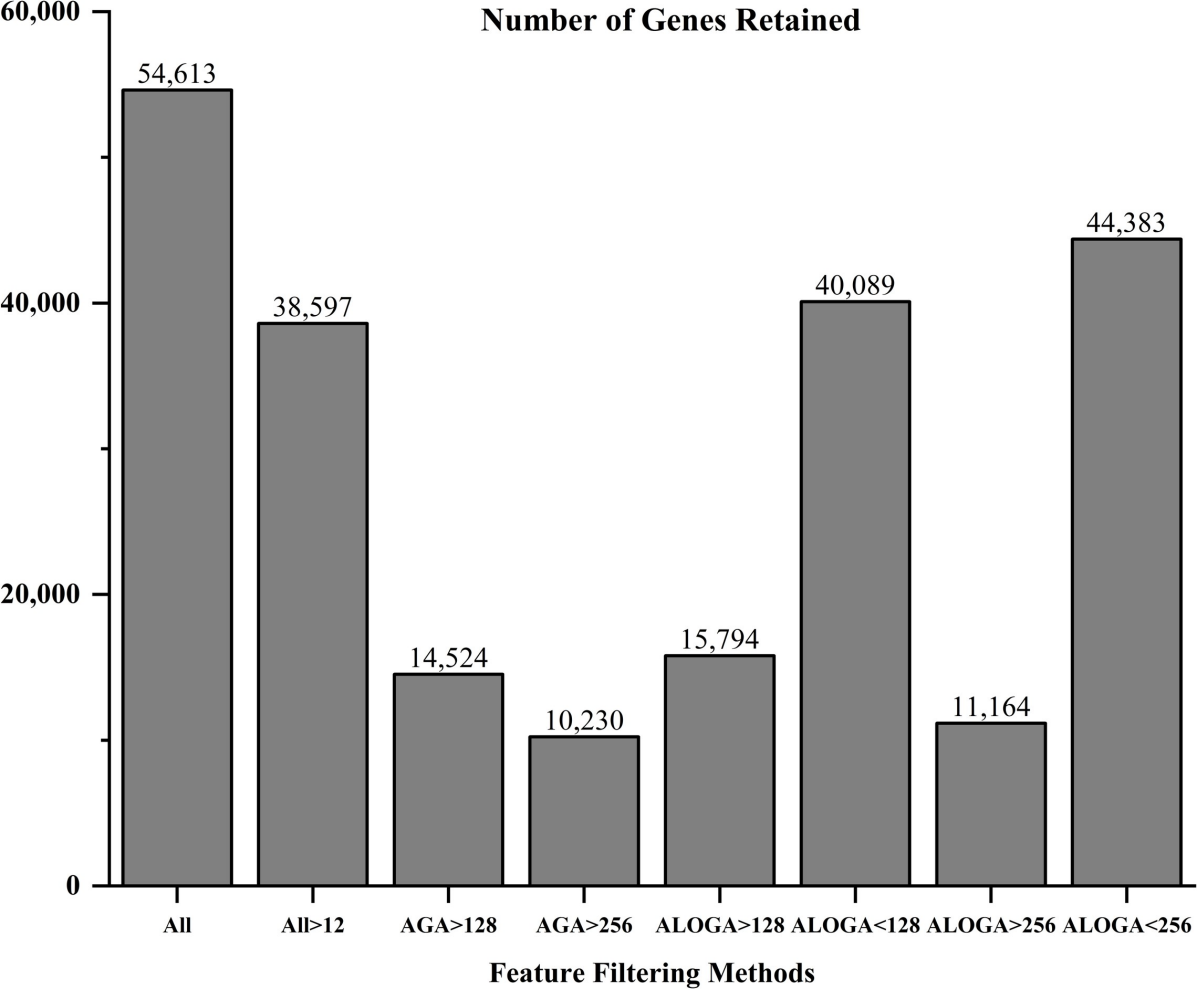
Number of genes retained after applying different feature filtering methods including: keeping all genes (All); genes with average expression values larger than 12 (All>12), a value detected by the IQR threshold detection method; genes with All PAH Group Average expression values larger than 128 (AGA>128) or 256 (AGA>256); genes with At Least One PAH Group Average expression value larger than 128 (ALOGA>128) or 256 (ALOGA>256); as well as with At Least One PAH Group Average expression value smaller than 128 (ALOGA<128), or 256 (ALOGA<256).

The overall data analysis scheme/workflow is presented in Fig 1. Without using any filtering routine that is commonly embedded in many microarray software packages for duplication removal and high IQR probe retention, our preliminary filtering methods resulted in dramatic differences in the number of retained probe sets as indicated in Fig 2. Particularly, removing genes with at least one group average smaller than 256 (or synonymously AGA>256) yielded only 10,230 features, which is 18.7% of the entire gene count from the raw microarray results. Likewise, if only genes with at least one group average smaller than 256 were selected (ALOGA<256), 44,383 genes would be retained, accounting for 81.3% (equals 1–18.7%) of the entire gene count. For comparison purposes, the conventional IQR-detected threshold filtering (All>12, which corresponds to probe-sets with expression profile IQR > 0.3) allowed 38,597 genes pass the screening process [13,14].

For each combination of classification tasks and feature filtering methods, we investigated the modeling performance synthesizing results from repeated MCCV, including feature ranking, model training and testing. Furthermore, average classification accuracies were reported based on the number of ranked features (refined or unrefined) used by each model that could lead to the lowest error rate (or synonymously, the highest accuracy). Again, we anticipated that this number (number of ranked features providing the greatest accuracy) could be highly variable depending on the nature of the classification task *per se* as well as the feature ranking and classification algorithms used under each routine.

### Multiclass classification for distinguishing each individual PAH patient group

**Fig 3:**
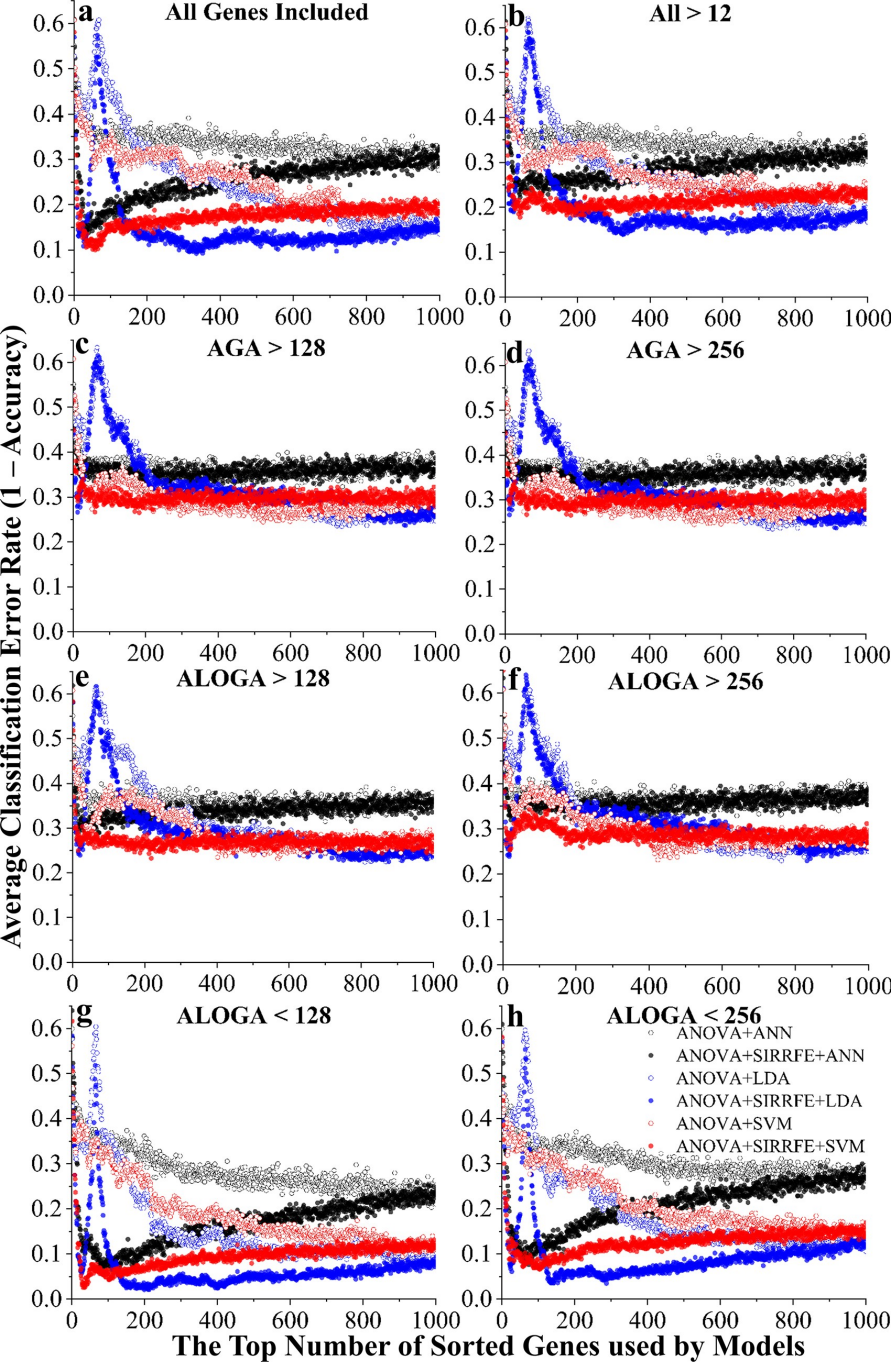
Classification error rate (1-accuracy) *vs*. the top number of ranked genes used by models, constructed to distinguish each individual PAH patient group. Different feature filtering methods were used including: (a) keeping all genes (**All**); (b) genes with average expression values larger than 12 (**All>12**), a value detected by the IQR threshold detection method; genes with **A**ll PAH **G**roup **A**verage expression values **larger than 128 (**c, **AGA>128**) or **256** (d, **AGA>256**); genes with **A**t **L**east **O**ne PAH **G**roup **A**verage expression value **larger than 128 (**e, **ALOGA>128)** or **256 (**f, **ALOGA>256)**; as well as with **A**t **L**east **O**ne PAH **G**roup **A**verage expression value **smaller than 128** (g, **ALOGA<128**), or **256** (h, **ALOGA<256**). Additionally, six feature ranking and classification routines were used, including: a simple one-way **A**nalysis of **V**ariances (**ANOVA**) method followed by **L**inear **D**iscriminant **A**nalysis (**ANOVA+LDA**), **S**upport **V**ector **M**achine-based (**ANOVA+SVM**), or **A**rtificial **N**euron **N**etwork-based (**ANOVA+ANN**) classifications; with or without incorporation of a novel **S**liced **I**nverse **R**egression-based **R**ecursive **F**eature **E**limination algorithm (**SIRRFE**).

Fig 3 depicts the error rate (measured as the number of miss-predicted patients divided by the total number of patients) change when more genes from the ranked feature list were included in the prediction model. Solid or empty markers indicate algorithms incorporating SIRRFE-based refinement, or without, respectively. Edge color differences indicate different classification algorithms (black-ANN, blue-LDA, and red-SVM).

Regardless of incorporating SIRRFE or not, LDA-based classification algorithms always resulted in an error peak across all feature filtering methods. Using very few (less than 50) ranked genes, both SIRRFE-refined SVM and LDA algorithms produced lower error rates compared with other data processing routines. Generally speaking, the ANN-based algorithms were not as accurate as the other two. The enhanced modeling performance, particularly in the top 200 genes range, caused by SIRRFE were extremely significant under All Genes (Fig 3A), All>12 as identified by the IQR method (Fig 3B), ALOGA<128 (Fig 3H), and ALOGA<256 (Fig 3H) filtering methods; and moderately significant under ALOGA>128 (Fig 3E) and ALOGA>256 (Fig 3F) methods. Across all feature filtering methods, it was greatly evident that both ALOGA<128 (Fig 3G) and ALOGA<256 (Fig 3H) had resulted in incomparably superior performance than others. Both filtering methods provided very small error rates using very few ranked genes (less than 50). Additionally, the ALOGA<128 (Fig 3G) filtering method appeared to be the optimal. SIRRFE refined SVM routines provided stable and satisfactory performance across all filtering methods. Additionally, SIRRFE refined LDA routines produced superior results around the top 370 genes range compared with SVM, but used substantially greater number of features. Interestingly, both AGA>128 (Fig 3C) and AGA>256 (Fig 3D) filtering methods tended to produce the worst results (>0.24 error rates), followed by both ALOGA>128 (Fig 3E) and ALOGA>256 (>0.23 error rates, Fig 3F). It was also noteworthy that it seemed inclusion of more ranked genes in those non-SIRRFE refined routines could always boost performance; however, adding more than 200 SIRRFE refined ranked genes into different classification algorithms could lead to little or even compromised performance. This accented the efficiency and effectiveness of our SIRRFE-based feature ranking/selection algorithms.

### Binary classification-the healthy control *vs*. the combination of HPAH and IPAH

**Fig 4:**
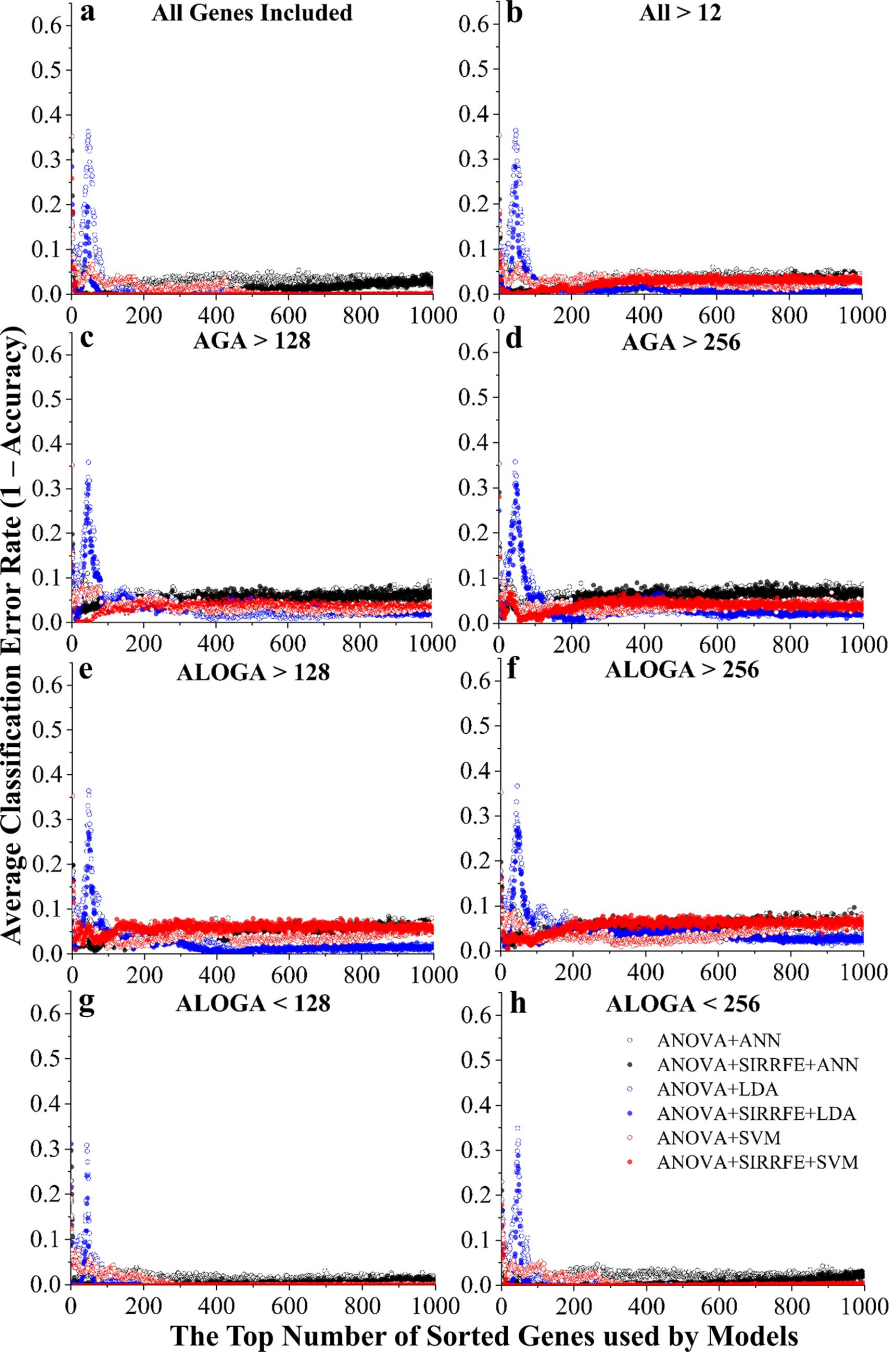
Classification error rate (1-accuracy) *vs*. the top number of ranked genes used by models, constructed to distinguish the healthy control *vs*. the combination of HPAH and IPAH patient groups. Different feature filtering methods were used including: (a) keeping all genes (**All**); (b) genes with average expression values larger than 12 (**All>12**), a value detected by the IQR threshold detection method; genes with **A**ll PAH **G**roup **A**verage expression values **larger than 128 (**c, **AGA>128**) or **256** (d, **AGA>256**); genes with **A**t **L**east **O**ne PAH **G**roup **A**verage expression value **larger than 128 (**e, **ALOGA>128)** or **256 (**f, **ALOGA>256)**; as well as with **A**t **L**east **O**ne PAH **G**roup **A**verage expression value **smaller than 128** (g, **ALOGA<128**), or **256** (h, **ALOGA<256**). Additionally, six feature ranking and classification routines were used, including: a simple one-way **A**nalysis of **V**ariances (**ANOVA**) method followed by **L**inear **D**iscriminant **A**nalysis (**ANOVA+LDA**), **S**upport **V**ector **M**achine-based (**ANOVA+SVM**), or **A**rtificial **N**euron **N**etwork-based (**ANOVA+ANN**) classifications; with or without incorporation of a novel **S**liced **I**nverse **R**egression-based **R**ecursive **F**eature **E**limination algorithm (**SIRRFE**).

Comparing all classification algorithms used in this task, both ANN and SVM yielded very similar performance, and both appeared to be better algorithms than LDA as indicated Fig 4. This was particularly obvious when very small quantity of ranked genes were used (<50). Regardless feature filtering methods, SIRRFE provided little improvement when LDA algorithm was used. Furthermore, under AGA>256 (Fig 4D), no improvement was observed across all three classification algorithms. However, SIRRFE generally enhanced model classification performance of both ANN and SVM-based algorithms. We also found similar error peaks while using LDA-based algorithms as reported in the previous session. Likewise, we observed that different feature filtering methods could have tremendous impact on the modeling results. In this binary classification case, we found that ALOGA<128 (Fig 4G), ALOGA<256 (Fig 4H), and no filtering (Fig 4A) could all provide very low error rates. Particularly, ALOGA<128 (Fig 4G) and 256 (Fig 4H) generated perfect classification (zero error rate) using less than 20 genes. Additionally, the majority of classification routines reached optimal accuracy within the top 100 genes ranking range, and the performance enhancement for including more genes diminished rapidly.

### Binary classification-HPAH *vs*. UMC

**Fig 5:**
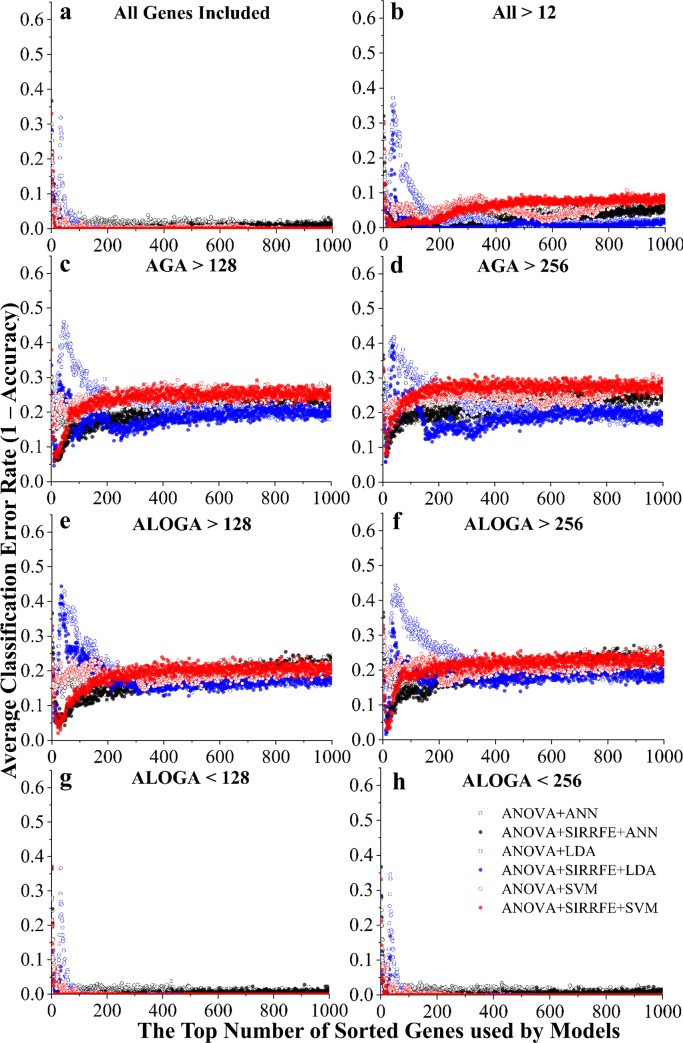
Classification error rate (1-accuracy) *vs*. the top number of ranked genes used by models, constructed to distinguish the HPAH *vs*. UMC patient groups. Different feature filtering methods were used including: (a) keeping all genes (**All**); (b) genes with average expression values larger than 12 (**All>12**), a value detected by the IQR threshold detection method; genes with **A**ll PAH **G**roup **A**verage expression values **larger than 128 (**c, **AGA>128**) or **256** (d, **AGA>256**); genes with **A**t **L**east **O**ne PAH **G**roup **A**verage expression value **larger than 128 (**e, **ALOGA>128)** or **256 (**f, **ALOGA>256)**; as well as with **A**t **L**east **O**ne PAH **G**roup **A**verage expression value **smaller than 128** (g, **ALOGA<128**), or **256** (h, **ALOGA<256**). Additionally, six feature ranking and classification routines were used, including: a simple one-way **A**nalysis of **V**ariances (**ANOVA**) method followed by **L**inear **D**iscriminant **A**nalysis (**ANOVA+LDA**), **S**upport **V**ector **M**achine-based (**ANOVA+SVM**), or **A**rtificial **N**euron **N**etwork-based (**ANOVA+ANN**) classifications; with or without incorporation of a novel **S**liced **I**nverse **R**egression-based **R**ecursive **F**eature **E**limination algorithm (**SIRRFE**).

Overall, the impacts of various feature filtering methods on the model performance significantly outweighed that attributed to different classification algorithms as indicated in Fig 5. Apparently, any kind of “denoising” filtering method [e.g. AGA>128, 256 (Fig 5C and 5D); All>12, (Fig 5B); or ALOGA>128,256 (Fig 5E and 5F)] had caused increased error rates. Additionally, both ALOGA<128 (Fig 5G) and ALOGA<256 (Fig 5H) as well as All-Genes-Included (Fig 5A) methods provided superb results (zero error) under all classification algorithms. This effect became even more pronounced when SIRRFE was incorporated. In general, all algorithms worked equally well under ALOGA<128 (Fig 5G), ALOGA<256 (Fig 5H), and All-Genes-Included methods (Fig 5A); except for the similar error peak induced by LDA as reported in the other classification tasks described preciously. Again, we observed significant performance boost using SIRRFE. One finding that was very unique for this classification task was that inclusion of more (>50) ranked genes in our classification routine had significantly increased error rates except for those high-performance filtering methods [e.g. ALOGA<128, 256, and All (Fig 5A, 5G and 5H)].

### Optimal model analysis

As expected, the multiclass classification for distinguishing each individual PAH patient group was the most challenging classification task, which required larger number of genes for achieving acceptable accuracies and the final outcome depended greatly on the type of classification algorithms used. Additionally, the performance enhancement using SIRRFE was very significant. The binary classification of the healthy control *vs*. the combination of HPAH and IPAH seemed to be much easier, with the majority of models yielding zero error rates using less than 20–30 genes. The contributions of different classification algorithms and SIRRFE refinement were not as great as those filtering methods. Finally, the binary classification task involving HPAH *vs*. UMC appeared to be the most responsive towards different feature filtering methods, with significant advantages observed under ALOGA<128, 256, or All-Genes-Included methods.

To further examine the optimal models that provided the lowest error rates (highest accuracy) using the least number of ranked genes, we provided a detailed summarization in Table 1. Additionally, considering that SIRRFE generally resulted in improved performance as indicated in Figs 3–5, we only reported the optimal SIRRFE-refined results for simplicity purposes. For the multiclass classification (Control *vs*. IPAH *vs*. HPAH *vs*. UMC), our best routine (ALOGA<128 with ANOVA+SIRRFE+LDA) achieved an accuracy value of 0.98 using 199 ranked genes. On average, ALOGA<128 appeared to be the optimal filtering method (average accuracy = 0.96) and LDA algorithm seemed to be the best classification algorithm without considering the numerical stability and the error peak observed in Fig 3. For the binary classification of the healthy control *vs*. the combination of HPAH and IPAH, the majority of the routines yielded perfect accuracies. Therefore, judging from the number of ranked genes for achieving the perfect prediction, both SIRRFE-refined LDA and SVM algorithms with ALOGA<128 filtering provided perfect accuracy using only eight genes. Overall, the ALOGA<128 filtering method appeared to be the optimal (average accuracy = 1, average number of ranked genes used = 11) and SVM seemed to be the best classification algorithm (average accuracy = 0.99, average number of ranked genes used = 19). Finally, the optimal model for the binary classification of HPAH *vs*. UMC was obtained using the ALOGA<128 filtering method and the SIRRFE-refined SVM algorithm, providing a perfect classification accuracy using only 10 ranked genes. On average, the ALOGA<128 filtering method appeared to be the optimal (average accuracy = 1, average number of ranked genes used = 18) and both LDA and SVM seemed to be good classification algorithm (average accuracy = 0.97, average number of ranked genes used = 14–16). Again, we thought SVM should be considered superior over LDA due to the error peak observed under LDA as indicated in Fig 5.

**Table 1:** The maximum accuracy (lowest error rate) with the least number of ANOVA-ranked genes achieved by different feature filtering methods and classification algorithms refined by SIRRFE under various classification tasks, including: the multiclass classification for distinguishing each individual PAH patient group (Control *vs*. IPAH *vs*. HPAH *vs*. UMC), binary classification of the healthy control *vs*. the combination of HPAH and IPAH, and the binary classification of HPAH *vs*. UMC.

**Goal 1: Control *vs*. IPAH *vs*. HPAH *vs*. UMC**										
			**The Maximum Accuracy (Number of Ranked Genes used) with Different Gene Filtering Methods**							**Average**
		All Genes	All > 12	AGA>128	AGA>256	ALOGA>128	ALOGA>256	ALOGA<128	ALOGA<256	
**Classification Algorithms**	**LDA**	0.91 (335)	0.86 (325)	0.76 (917)	0.76 (794)	0.75 (876)	0.77 (832)	0.98 (199)	0.97 (287)	0.84 (570)
	**SVM**	0.9 (58)	0.82 (41)	0.73 (182)	0.72 (8)	0.77 (218)	0.75 (19)	0.97 (31)*	0.93 (104)	0.82 (82)
	**ANN**	0.87 (32)	0.77 (41)	0.69 (191)	0.66 (221)	0.71 (43)	0.68 (22)	0.94 (87)	0.92 (74)	0.78 (89)
**Average**		0.89 (142)	0.82 (135)	0.73 (430)	0.71 (341)	0.74 (379)	0.73 (291)	0.96 (106)	0.94 (155)	0.81 (247)
**Goal 2: Control *vs*. (IPAH + HPAH)**										
**Classification Algorithms**	**LDA**	1 (13)	1 (16)	0.99 (12)	0.99 (188)	0.99 (419)	0.99 (18)	1 (8)*	1 (18)	0.99 (86)
	**SVM**	1 (13)	1 (13)	1 (13)	0.99 (56)	0.99 (9)	0.99 (21)	1 (8)*	1 (12)	0.99 (18)
	**ANN**	1 (24)	1 (14)	1 (13)	0.99 (55)	0.99 (62)	0.99 (19)	1 (16)	1 (27)	0.99 (29)
**Average**		1 (17)	1 (14)	0.99 (13)	0.99 (100)	0.99 (163)	0.99 (19)	1 (11)	1 (19)	0.99 (45)
**Goal 3: HPAH *vs*. UMC**										
**Classification Algorithms**	**LDA**	1 (22)	1 (130)	0.95 (10)	0.94 (10)	0.94 (13)	0.98 (11)	1 (15)	1 (16)	0.97 (28)
	**SVM**	1 (17)	1 (14)	0.94 (11)	0.92 (13)	0.98 (22)	0.97 (15)	1 (10)*	1 (23)	0.97 (16)
	**ANN**	1 (49)	1 (49)	0.94 (12)	0.92 (16)	0.96 (25)	0.95 (19)	1 (30)	1 (35)	0.96 (30)
**Average**		1 (29)	1 (64)	0.94 (11)	0.93 (13)	0.96 (20)	0.97 (15)	1 (18)	1 (25)	0.97 (25)

*Indicates the optimal routine (filtering method + classification algorithm) evaluated based on accuracy (the greater the better) and the number of ranked genes used (the smaller the better).

Finally, similar to what was reported in Figs 3–5, the binary classification of the healthy control *vs*. the combination of HPAH and IPAH seemed to be the easiest task, providing a very high accuracy (overall average = 0.99) using a small number of ranked genes (overall average = 49). The multiclass classification (Control *vs*. IPAH *vs*. HPAH *vs*. UMC) was the most challenging task with an overall accuracy of 0.81 using 247 genes on average. Across all classification goals/tasks, the ALOGA<128 filtering method consistently provided the highest accuracy with the least number of genes utilized.

### Gene expression analysis

**Fig 6:**
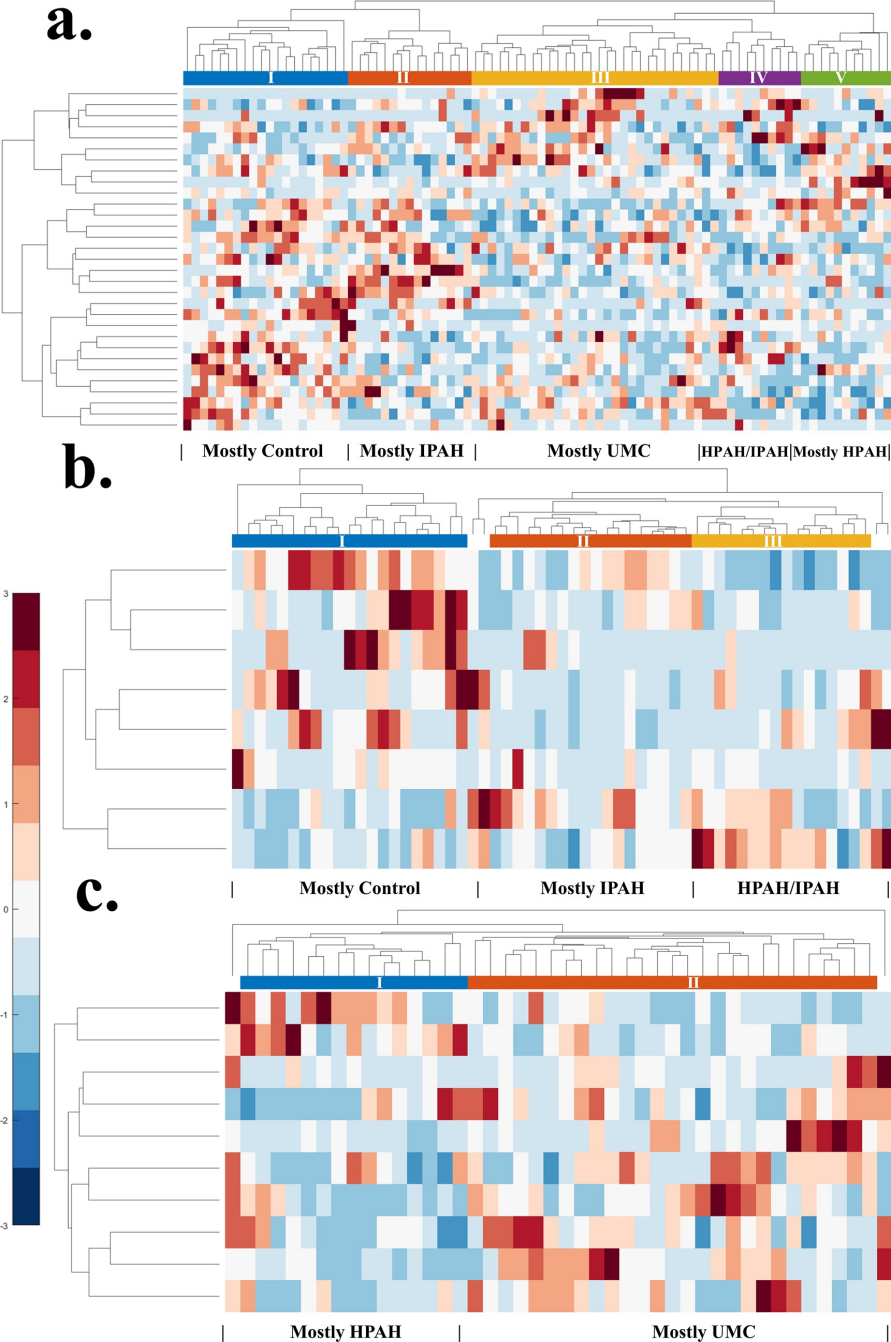
Conventional unsupervised hierarchical clustering indicated poor patient group separation compared to machine learning-based models. Clustered heat maps were generated using (a) the identical 31 ranked genes used for constructing the support vector machine-based (SVM) multiclass classification model, which distinguishes each individual PAH patient group (Control *vs*. IPAH *vs*. HPAH *vs*. UMC) with an accuracy value of 0.97; (b) the identical eight ranked genes used for constructing SVM-based classification model, which distinguish the healthy control *vs*. the combination of HPAH and IPAH with a perfect accuracy; and (c) the identical 10 genes used for constructing the SVM-based classification model, which separates HPAH *vs*. UMC with a perfect accuracy All expression levels are standardized along the row dimension and the hierarchical cluster trees are generated using unsupervised linkage functions based on the inner squared/unweighted average distance between all pairs of objects in any two clusters. Clustering of patients are depicted by the dendrogram on top with cluster numbers labeled in Latin numbers. Higher expression levels are represented as increasing red, lower ones are represented as increasing blue, and white represents close to the average. All gene names are appended to the right of the map with clustering dendrogram appended to the left. Detailed clustering of patient expression profiles, including each patient’s PAH status and gene probe identifications information, was included at S1 Fig.

To further analyze the refined list of highly informative genes (Table 1) used for constructing the optimal classification models, and to evaluate the possibilities of distinguishing patient groups without relying on advanced machine learning algorithms for comparison purposes; we also performed conventional unsupervised hierarchical clustering that is commonly used in microarray data analysis. Using this method, genes that share similar expression patterns are more likely to be clustered together. Particularly, we selected the identical groups of genes used for constructing the optimal machine learning classification model under each task (Table 1). For the first classification task (Control *vs*. IPAH *vs*. HPAH *vs*. UMC), SVM-based algorithm achieved an accuracy value of 0.97 using only 31 filtered (ALOGA<128) and SIRRFE-refined genes (Table 1). The LDA-based model provided slightly better accuracy (0.98) under the same filtering method, but used substantially larger number of genes (199), thus, was not considered optimal. Instead of using SVM-based algorithms, unsupervised hierarchical clustering identified five groups of patients based on the expression pattern of these 31 genes. As indicated in Fig 6A, Group I patients primarily belonged to the Control group. Group II, III, and V consisted mainly of patients from the IPAH, UMC, and HPAH groups, respectively. Group IV had a mixture both IPAH and HPAH patient groups. From a genetic expression perspective, it appeared that certain protein coding genes such as the KIAA1217, TNFRSF25, ADCY5, NFIA (FLJ39164), and LHFP; as well as some non-protein coding genes, such as the LINC01181 (FLJ10489) typically had relatively higher expression values in the control patient groups than others. Genes such as the CARD19 (c9orf89), PIK3C3, LINC00308 (C21orf74), SLC7A5P1 (LAT1-3TM), BDNF, NECAB1 (EFCBP1), ZNF221 usually had higher expression values in the IPAH patient group. The LINC00461 (LOC645323), DACH1, TXLNGY (CYORF15A), CADPS, SSTR2, ZNF335 genes all had relatively higher expression values in the UMC patient group. According to the KEGG pathways database [15], PIK3C3 is greatly involved in the phosphatidylinositol signaling system, SSTR2 is involved in the cAMP signaling pathway and the neuroactive ligand-receptor interaction processes, and BDNF is involved in the neurotrophin signaling pathway. For the second classification task (control *vs*. the combination of HPAH and IPAH), both LDA and SVM-based algorithms under the ALOGA<128 filter provided perfect accuracies using only eight SIRRFE-refined genes (Table 1). Using the identical list of genes and unsupervised hierarchical clustering method, three major patient clusters could be identified as Group I dominated by Control-group patients; Group II, predominately IPAH patients; and Group III, mainly HPAH patients as indicated in Fig 6B. Additionally, each individual gene behaved differently among different patient groups. Both LHFP and FTCD genes seemed to have greater expression values in the Control patient group than the other two. For the last classification task (HPAH *vs*. UMC), SVM-based model achieved perfect accuracy using 10 genes filtered by the ALOGA<128 method (Table 1). Unsupervised hierarchical clustering method identified two patient clusters using the same 10 genes, including Group I consisting of mainly HPAH patients, and Group II mainly UMC patients as indicated in Fig 6C. Both PLLP and SGCA genes had greater expression values in HPAH patient groups than UMC. LEF1 genes indicated drastically increased expression levels in certain UMC patients than HPAH. Other genes lacked consistency in expression values between patient groups, such as CYP21A2 and SYNE2. To summarize, all unsupervised hierarchical clustering models yielded very limited performance compared to machine learning-based models, which typically offered near-perfect/perfect accuracies.

**Fig 7:**
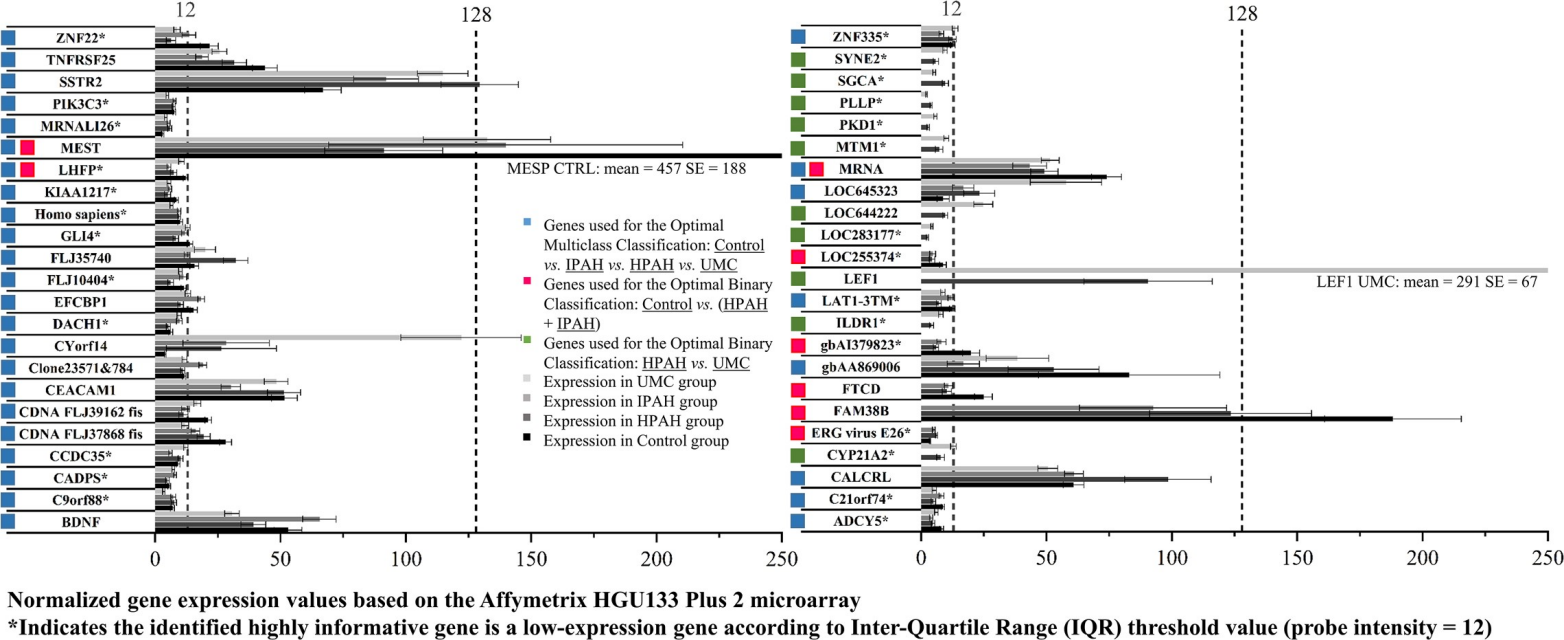
Expression values and standard errors of genes within each patient group under the optimal feature filtering method (ALOGA < 128) used for (blue bars) distinguishing each individual PAH patient group (Control *vs*. IPAH *vs*. HPAH *vs*. UMC) with an accuracy value of 0.97; (red bars) distinguishing the healthy control *vs*. the combination of HPAH and IPAH with a perfect accuracy; and (green bars) distinguishing the HPAH *vs*. UMC with a perfect accuracy. Genes labeled using more than one color bar indicate sharing by multiple classification tasks. Additionally, the group averages of the UMC patient group, as well as the Control and IPAH groups were not displayed under the red bar and the green bar labelled genes, respectively; because they are not used in these classification tasks.

Finally, Fig 7 summarized the average gene expression values and their corresponding standard errors within each patient group under the optimal feature filtering method (ALOGA < 128) as indicated in Table 1. The most remarkable finding was that out of the total 46 genes used by the optimal models across all three classification tasks, only four genes had at least one group average expression value larger than 128 (SSTR2, MEST, LEF1, and FAM38B). In other words, more than 91% of the highly informative genes used for constructing the optimal models had all group averages less than 128. Additionally, each classification routine tended to generate a separate list of features. Only three genes were shared between tasks 1 and 2, including LHFP, MRNA, and MEST. Last but not the least, 27 out of the total 46 genes (60%) had probe intensity values smaller than 12, a threshold identified by the IQR filtering algorithm.

Original array data are available at S1 Appendix and S2 Appendix. IQR-based threshold detection report, codes and detailed modeling results files are made openly available at S3 Appendix. Detailed clustering of patient expression profiles (Fig 6), including each patient’s PAH status and gene probe identifications information, is presented in S1 Fig.

## Discussion

In this study, we designed and implemented a novel and efficient data analysis paradigm using high-quality clinical data obtained from cultured, unaffected tissues that are free of contamination from drug effects with well-controlled confounding influence from other pathogenesis/molecular factors. The reason for using cultured blood samples as a proxy for evaluating PAH etiology is three-folds. First, the genetic expressions of fresh blood cells are largely impacted by drug effects, the inflammatory state in the lungs of PAH patients, and by other factors induced by individual variations. These factors could entirely override the impacts of baseline genetic differences (particularly on those low-expression genes), and eventually make them invisible. On the other hand, variations in gene expression in these cultured peripheral blood mononuclear cells are free of contamination and also reflect the key underlying differences in gene expression, particularly related to PAH, which is true across tissue types in while-body metabolism and has been verified by our group in several previous studies [16,17]. Second, bone-marrow derived cells are found to be more essential to many disease processes. Particularly, transplantation of patient-derived bone marrow has been enough to induce PAH in mice [18], and transplantation of mutant mice with wild-type bone marrow ameliorates it [19] in more than one model [20]. Therefore, these cultured blood cells actually have important etiological roles in identifying PAH.

To directly test the hypothesis that low-expression genes might be greatly informative in differentiating different forms of PAH patient groups, we proposed a series of feature filtering methods including a traditional IQR-based filtering method and others that are rarely used in conventional microarray-based clinical studies. As a matter of fact, information relating to adopting similar approaches in other disease studies of human being or other organisms is extremely limited, even non-exist. Again, this is because the majority of these low-expression genes are typically removed manually by the researchers or by certain “denoising” algorithms or feature filtering functions offered by many software packages (e.g. IQR) as part of the standard data analysis pipeline. Furthermore, in this study, we also formalized a novel feature ranking/selection algorithm (SIRRFE), which provided excellent modeling performance when incorporated with state-of-art machine learning algorithms. Several findings could provide important insights into designing innovative algorithmic approaches for analyzing microarray data and enhance our understanding on the molecular pathogenesis of PAH and its interrelationship with BMPR2 mutation.

Some highlights from this research finding are described in the following. First, as indicated in Fig 2, our most efficient gene filtering method (ALOGA < 128) only resulted in a small reduction in the number of probe sets selected (26% less). However, the performance boost was quite remarkable as presented in Figs 3–5. This was consistently observed across all filtering methods as indicated in Figs 3–5, but the most obvious effect was found in Fig 5. This indicated that there exist a decent portion of genes that have high expression values across all patients but are indeed less informative in identifying different PAH patient groups. Likewise, genes with at least one PAH patient group average less than 128 (or 256), including those with all group averages less than 128 (or 256), could actually serve as important signature features for constructing accurate and robust classification models. Removing these genes could cause significant losses in feature number (Fig 2) and increased error rates as observed under AGA>128 and AGA>256 (Figs 3–5). Generally speaking, the more low-expression genes (<12 or 128) removed from the dataset, the less accurate the models became. This was consistently observed in Figs 3–5 as indicated by the increased error rates from All genes, All>12, AGA>128 to AGA>256. This, again, indicated that low-expression genes are indeed important. Particularly, even the conventional IQR-based low-expression gene filtering could result in elimination of highly informative low-expression genes. Therefore, extra caution should be used while adopting various feature filtering methods/algorithms. To date, the main-trend microarray-based data analysis still focuses on the post-filtering stage (e.g. t-test, correction methods, p-value, etc.), but our study highlighted that the importance of pre-analysis filtering methods/algorithms should never be overlooked. Second, our SIRFE-based feature refining algorithm resulted in a significant reduction in error rate across several filtering methods and classification algorithms. Particularly, it became extremely effective once incorporated with more complex non-parametric algorithms, such as the ANN and SVM. The general superior performance of SVM than ANN could be caused by the incorporation of kernel methods and the uncertainties associated with the optimal ANN structure setting (e.g. number of hidden layers, number of neurons, types of excitation functions, etc.). This finding could potentially lay the essential groundwork for future work in the bioinformatics domain, where information abounds with using state-of-the-art algorithms such as ANN, SVM, deep learning, etc.; but efficient feature ranking/selection algorithms is always lacking. Third, the LDA-based algorithms always resulted in an error peak around the top 50 to 100 ranking feature region. We attributed this primarily to the functionality limitation of LDA itself because no other classification methods had produced similar trends. Last but not the least, all SIRRFE refined algorithms had produced very impressive optimal accuracy values (overall average 0.93) across various combinations of gene filtering methods and classification algorithms as indicated in Table 1. However, the number of genes used for achieving these optimal accuracy values varied greatly. This finding again accented the effectiveness of SIRRFE. More importantly, the ability for generating highly accurate classification/prediction models with only 8 to 31 features based on cross validated datasets could lead to the development of clinical analysis software packages for future use. We expect that similar methodology, model construction and data analysis routines could be extended to other pathological domains besides PAH.

The clustered heat maps (Fig 6A–6C) of all the three classification tasks provided an overview of the general patterns of the gene expression and sample grouping across the 86 patients using conventional unsupervised hierarchical clustering methods. It was noteworthy that the gene lists used in the clustering analysis were filtered and refined by SIRRFE, and were identical to those used in advanced machine learning models (e.g. SVM and LDA) indicated in Table 1. However, unlike SVM or ANN, which transformed original data using kernel tricks or abstract neural network structure to achieve near-perfect classification; conventional unsupervised hierarchical clustering failed to separate patient groups accurately even with filtered and SIRRFE-refined genes (Fig 6). Therefore, the chances for achieving great classification performance using unsupervised clustering methods with unfiltered/unrefined gene lists are extremely low. Additionally, it was noteworthy that none of the filtering nor SIRRFE algorithms depended on any classification model. Therefore, there were no biases towards any classification method. As indicated in this study, the overall performance of conventional heat map-based microarray data analysis was extremely limited, particularly when solving complex data analytic problems. Due to the fact that many informative genes identified in each task were low-expression genes, information relating to their functionality and molecular pathways are extremely scanty. Some highlights included KIAA1217 genes identified to be highly informative for completing the first, also the most challenging classification task. It was recently found as a novel Rearranged-during-Transfection fusion gene detected in a small portion of lung adenocarcinomas and was known as an oncogenic driver factor [21]. Another important gene identified in task 1, Dachshund 1 (DACH1) was found as an inhibitor that could prevent proliferation and invasion of lung adenocarcinoma cells as well as growth of tumor cells through repression of other factor genes [22]. For both task one and two (control *vs*. the combination of HPAH and IPAH), the mesoderm-specific transcript (MEST) gene, also known as the paternally expressed gene 1, was recognized as of high importance. It was found to be correlated with frequent loss of imprinting in lung adenocarcinoma cases [23,24]. For the last classification task (HPAH *vs*. UMC), the lymphocyte enhancement factor 1 (LEF1) gene, which plays important roles in mediating lung tumor occurrence [25,26], had relatively greater expression values than others. Additionally, the 21-hydroxylase instruction gene (CYP21A2), another highly informative gene for task three, was recently found in the developing distal epithelium of the human developing lungs, potentially with its products binding to the glucocorticoid receptor and exert certain intracrine actions [27].

There are some potential limitations associated with this study. First of all, only a limited number of machine learning algorithms were used in the model construction and validation processes. We expected this shouldn’t affect the result interpretation much because those selected ones represent some of the most popular methods in data science domain. Additionally, the main goal of this study was to test the hypothesis stating that clusters of low-expression genes might collectively control PAH, thus, model construction procedure *per se* was not as important as obtaining the optimal models and the identification of the most informative gene list/ranking. Third, because the primary goal of this study was to identify a comprehensive ranking list of genes that could be uniformly evaluated using different gene filtering and classification algorithms, no separate initial feature ranking method was used for each data analysis routine. However, our SIRRFE-based gene refining algorithms was carried out independently within each classification method. Fourth, our optimal model analysis was based on the minimal MCCV error. We noted that the MCCV process involves randomness. Other models, especially those with gene numbers similar to the optimal models, might have MCCV errors not statistically different from the optimal models. In other words, one may derive a different optimal model by running the same program with different random seeds. However, provided the models were built and selected by using the same gene ranking list, we would expect they share sufficient overlap and the change of optimal models should not affect our primary findings much. Fifth, our approach used kernel method-based classification algorithms as a proxy for evaluating the importance of all genes used in each task. The underlying methods considered the interactions and correlations among all features, but these interactive relationships were not explicitly modeled or presented in this work, emphasizing the necessity for using interactive component modeling/selection methods in future research [28]. Sixth, adding another set of independent validation cohort could greatly strengthen the study. However, due the lack of publicly available high quality datasets, it is not possible to validate these machine learning models on other datasets at the present time. The particular dataset used in this study is unique; because it is obtained from cultured, unaffected, and uncontaminated tissues. The underlying genetically based differences in gene expression are easily overwhelmed by effects of end-stage diseases and treatments (e.g. prostacyclin) as those obtained from fresh isolated blood samples, making them unusable for this kind of tasks. Meanwhile, we also recognize that the cell immortalization processes, which should keep cross-group comparisons valid, might mask or enlarge certain differences in gene expression. However, the baseline genetic differences should be preserved and captured by our data analysis. We felt that the standard cross-validated modeling paradigm (e.g. MCCV) should provide adequate independency and control over model over-fitting. Last but not the least, our work relied on the hypothesis that gene expression differences are essentially caused by genetic functional differences, thus, the captured differences were the cause of PAH instead of the consequences.

To date, microarray analysis has become an effective and powerful tool widely used by many scientists in PAH research, providing reliable detection of genome-wide expression differences among patient groups [6]. Information abounds with use of standard microarray data analysis pipeline, which involves background noise reduction and normalization followed by gene expression analysis using fold change values or *t*-statistics, as well as co-expression patterns detection using different clustering/scaling algorithms (e.g. principal component analysis). Additionally, the IQR-based filtering methods are still dominating, which involves great amount of empiricism and subjectivity in the q-value threshold selection processes [13,14]. Only a few studies focused on investigating the signal threshold of gene expression when analyzing microarray data, and almost all of them endorsed the removal of low-expression data. For example, Li et al. [12] adopted novel signal threshold algorithms, which greatly reduced false positive/negative rates compared with two-fold change methods. The algorithms incorporated a two-step filtration strategy, which was very similar to our feature filtering methods but using slightly different cutoff values (100 and 200). In another study, Aris et al. [11] performed sensitivity analysis on different probe set intensity extraction methods and found that the minimum intensity cutoff thresholds were the most influential parameter affecting performance, and low intensity genes should be eliminated for achieving better sensitivity. More importantly, the study explicitly emphasized the removal of low-expression noisy data using cutoff intensity value of 100, which produced relatively stable performance with reasonable false positive rate. The most important finding of this study was that a large portion of the highly informative genes were low-expressed, regardless of cutoff threshold identification methods (IQR-based or simple probe intensity value-based approaches). Thus, if conventional IQR-based methods were adopted, 60% of highly informative genes would be eliminated before reaching the phase for identifying differentially expressed genes. To date, information related to the evaluation and investigation of low-expression genes from microarray data is still extremely limited. Oftentimes, attention was given to how different filtering algorithms/methods could be used for effectively removing low-expression genes [29] or how signal intensity of these genes be enhanced [30]. For example, a previous study indicated that microarrays with long oligonucleotide probes could greatly improve signal intensity of low-expression genes, which might greatly influence the results [31]. Little research has evaluated the importance and/or the collective effects of low-expression genes on various animal and human diseases. Particularly, systematic modeling work focusing on PAH pathobiology is almost nonexistent. We believe similar molecular pathways and/or genetic mechanisms should also exist in the etiology of other types of diseases, and explicitly modeling the genetic signaling network and interplay could provide important insights into better understanding of the underlying mechanisms of the pathogenesis of PAH. These are not the main focuses of this study, but are indeed important future directions.

## Conclusion

In conclusion, this research indicated that low-expression genes, typically removed during the background de-noising processes when analyzing microarray data, possess important biological information for controlling PAH. Conservatively speaking, even though each individual low-expression gene might be of little importance, the clusters of many should provide significant synergetic impacts on many pathophysiological processes. Integrating the information provided by these clusters of highly informative low-expression genes with advanced machine learning algorithms and novel feature ranking/refining methods (e.g. SIRRFE) could help construct robust classification models that can accurately predict each patient’s PAH status using very few genes. Finally, we acknowledge that using other advanced sequencing method such as RNA-seq could lead to more important findings in novel pathway/biomarker identification and risk evaluation related to PAH [4,31,32], thus, should also be investigated using similar approaches as proposed in this study in the future.

## Methods

### Ethics statement

Human subject research ethnic statement is included in the manuscript and approved by the Vanderbilt University School of Medicine. Study protocols were approved by the VUMC Institutional Review Board. All participants had given written consent to participate in clinical studies and underwent genetic counseling according to guidelines established by the American College of Cardiology Foundation and the American Heart Association prior to blood sampling.

### Data preparation

All patient data were obtained from the Vanderbilt Pulmonary Hypertension Research Cohort (VPHRC), which houses more than 40 year of clinical and biologic specimens of patients, including those with IPAH and HPAH and their detailed medical history and family pedigree. Various forms of PAH mutations were detected particularly in the HPAH patient group, including frameshift, insertion/deletion, missense, and nonsense mutations. All PAH patients were diagnosed by specialist physicians at Vanderbilt University Medical Center (VUMC) or other regional hospitals. PAH was determined either by autopsy evidences indicating plexogenic pulmonary arteriopathy without alternative causes or by cardiac/clinical criteria widely accepted internationally [33]. Study subjects of VPHRC were recruited via the Vanderbilt Pulmonary Hypertension Center, the Pulmonary Hypertension Association, and the NIH Clinical Trials website (http://clinicaltrials.gov). Study protocols were approved by the VUMC Institutional Review Board. All participants had given written consent to participate in clinical studies and underwent genetic counseling according to guidelines established by the American College of Cardiology Foundation and the American Heart Association prior to blood sampling [34]. Ehylenediaminetetraacetic acid (EDTA) anticoagulated blood samples were carefully sampled at the time of hospitalization or clinical visits and then mailed via commercial blood shipping kit. Genomic DNA was isolated using the Puregene DNA Purification Kits (Gentra, Minneapolis, Minn.). Mutations of BMPR2 gene were performed by RT-PCR described previously, and the results were reported in a recent paper published by our research group [35]. We performed lymphocyte sampling/culturing from all patient groups using exactly the same protocols established in previously published article [16]. In particular, lymphocytes were isolated from anticoagulated whole blood within 48 hr of collection and were then exposed to Epstein-Barr Virus (EBV) to induce cell immortalization. Two ml blood was diluted with 2 ml Phosphate-Buffered Saline (PBS) solution, layered on top of 3 ml of Lympho Separation Medium (MP Biomedicals) and centrifuged for 10 minutes at 1,000 × G at room temperature. Using a Pasteur pipet, the lymphocytes were removed from the serum/Lympho Sep Media interface, washed in 10 ml PBS and then resuspended in 3 ml lymphoblast media (RPMI 1640 media containing L-glutamine, and 20% fetal bovine serum) containing 2 μg/ml cyclosporine. The lymphocytes were then infected with 3 ml Epstein-Barr virus (EBV) and transferred to a T-25 vent capped flask. The cells were incubated at 37°C/5% CO2 and fed weekly with lymphoblast media plus cyclosporine until signs of growth occurred. RNA was isolated from cultured lymphocytes using a Qiagen RNeasy mini kit (Valencia, Calif.). Complimentary DNA was synthesized and biotin-labeled complimentary RNA was produced by in-vitro transcription reaction. Affymetrix HGU133 Plus 2 microarrays (Affymetrix, Foster City, Calif.) were hybridized with 20 μg cRNA. Hybridization, washing, staining, and array probe scanning were carried out according to the protocol specified in the Affymetrix GeneChip Expression Analysis Manual.

### Datasets

The detailed patient characteristics data and microarray expression results analyzed using the Robust MultiArray Average algorithm (RMA) and R2.13/Bioconductor2.8 analysis were reported in a previous study conducted by our group [1]. In that study, we deliberately removed genes that have low-expression values according to common practices. For this study, we used the original background-corrected, normalized/summarized expression data processed by RMA function in the Affy Package. The summarized data contained 54,613 features (gene probes) for each of the 86 samples (patients), including 22 healthy control, 20 IPAH, 17 HPAH, and 27 UMC patients.

### Classification targets and preliminary filtering

One specific goal for the current research was to construct classification tools/models that could provide the maximum accuracy while predicting patients’ PAH status using the least amount of genes. Considering the fact that the complexity of the nature for the proposed tasks could greatly affect the performance, we established classification targets using the following grouping regime: 1) multiclass classification with each PAH group forming its own target group, 2) binary classification including the healthy control *vs*. the combination of HPAH and IPAH, 3) binary classification including HPAH *vs*. UMC; with the anticipated computational difficulty levels arranged in a descending order.

Again, we expected that some low-expression genes might collectively affect certain PAH pathogenesis pathways and were usually removed manually by the researchers or by the preprocessing algorithms (e.g. IQR) embedded in microarray software packages. In light of this, we used eight different feature filtering methods based on the RMA-normalized microarray data, including: using all genes; or genes with average expression values larger than 12 (All>12), a value detected by the IQR threshold detection method [13,14]; or using genes with all PAH group average expression values larger than 128 (AGA>128) or 256 (AGA>256); or genes with at least one PAH group average expression value larger than 128 (ALOGA>128) or 256 (ALOGA>256); or genes with at least one group average expression value smaller than 128 (ALOGA<128) or 256 (ALOGA<256). The rationale for this feature filtering regime is that: method 1 could preserve all expression data including those “noises”; method 2 serves as a comparison method commonly used by researchers, which removes low-expression genes based on IQR expression profile values; methods 3 and 4 closely imitated popular manual preprocessing algorithms, which treat low-expression genes as undependable/unreliable features; methods 5 and 6 would remove consistently low-expressed genes across all patient groups; and method 7 and 8 should capture the clusters of low-expression genes that might be greatly informative at distinguishing different PAH groups when functioning collectively.

### Feature ranking/selection and classification

For each classification task and preliminary filtering method combination (3 groupings × 8 filtering methods = 24 combinations) as indicated in the previous session, a standard workflow of feature ranking, selection, model construction and validation was carried out independently.

Firstly, we performed the one-way Analysis of Variance (ANOVA) for each gene and ranked the genes according to their p-values. A small p-value indicated that the mean gene expression values were significantly different between two groups, so that the gene could potentially serve as a good biomarker for distinguishing one group from the other. The smaller the p-value was, the more significant the differences were. Particularly, for each classification task, a different set of p-values were generated accordingly (four, two, and two groups for task one, two, and three; respectively). Note that one-way ANOVA was somewhat analogous to the correlation methods in two groups setting. It is simple and effective methods widely used for feature ranking. However, its major drawbacks is that it fails to generate compact gene sets and it may miss some complementary or highly correlated genes that have little contribution when functioning alone [36]. Thus, in our study, we retained the top 1000 genes based on the p-value (p-values < 0.001), which conserved the majority of highly informative genes for each classification task and effectively controlled the computational complexity before entering the feature refining step.

Secondly, we proposed a novel Sliced Inverse Regression-based Recursive Feature Elimination algorithm (SIRRFE) in this study to further refine the feature ranking list. Sliced inverse regression (SIR) is a popular dimension reduction method in multivariate statistics and has been used in many fields of science [37, 38]. It can be used in both regression and classification problems. For classification problem, it typically reduces the dimension of the data with *k* target groups (classes) down to the most *k-1* relevant dimensions by solving a generalized eigen-decomposition problem:

Γβ=λΣβ,

Where Γ is the between group covariance matrix and Σ is the covariance matrix of the whole data. The eigenvectors *β* associated to large eigenvalues are called effective dimension reduction (EDR) directions and could be used for transforming the original high dimension data to low dimensional data. Each reduced dimension becomes a linear combination of the original features. In this paper, we proposed an innovative recursive feature refining algorithm based on SIR, which used the magnitude of the coefficient associated each gene to rank its importance. Particularly, since our dataset has a very high dimensionality (54,613 in the case of using all genes or 1000 after preliminary selection after ANOVA) but limited sample size (86 patients), using SIR as a direct feature ranking method might greatly compromise the accuracy of the feature ranking; which is a classic problem called “curse of dimensionality,” well known in the machine learning community. However, the least informative feature could always be considered as less influential/informative and a recursive feature elimination (RFE) procedure could well overcome this problem [36,39,40]. Therefore, we proposed the following SIRRFE Algorithm:

Inputs:

Training samples: *X*_0_ = [*x*_*ij*_]_*i* = 1:*N*,*j* = 1:*G*_ with *N* being the number of patients and *G*_0_ the number of genes, class labels: y = [*y*_1_,*y*_2_,…*y*_*N*_]^*T*^, *k* = number of groups, feature ranking list r = [],

Initialize: *S* = 1: *G*_0_, *X* = *X*_0_,*G* = *G*_0_

Updates:

• Run SIR algorithm to obtain the EDR directions associated to the largest *l* = min(*G*,*k*−1) eigenvalues *β*_1_ = [*β*_11_,*β*_12_,…,*β*_1*G*_],*β*_2_ = [*β*_21_,*β*_22_,…,*β*_2*G*_],…, *β*_*l*_ = [*β*_*l*1_,*β*_*l*2_,…,*β*_*l*,*G*_]

• Find $g_{*}=arg\;\min_{1\leq g\leq G}\beta_{1,g}^{2}+\beta_{2,g}^{2}+\cdots +\beta_{lg}^{2}$

• *r* = [*S*(g_*_),*r*]

• *S* = *S*\*S*(g_*_)

• *X* = [*x*_*ij*_]_*i* = 1:*N*,*j*∈*S*_

• *G* = *G*−1

Repeated until: *S* = [] (or equivalently G = 0)

Outputs:

Updated feature ranking list r

Finally, we tested the diagnosis power of the genes that were refined by SIRRFE or not using three popular machine learning algorithms (LDA, SVM, and ANN) that have been widely used in a broad array of problem domains [41–46]. Specifically, we used the Statistics and Machine Learning Toolbox of Matlab Programming Language (The MathWorks, Inc., Natick, Massachusetts) for implementation. Additionally, we used 0.0001 to slightly regularize the covariance matrix in LDA to guarantee its invertibility when it is singular. For SVM, we adopted the one-versus-one coding design and applied linear kernel SVM for each binary classification problem. For ANN, we used five hidden layers in setting the neural network structure. Monte-Carlo cross validation (MCCV) technique was used in the model training and validating processes. Specifically, the entire dataset was randomly divided into two sections, with 80% of the samples retained for training the remaining 20% for testing. The classification model was then built using the training set and the testing set was used for prediction and performance evaluation. This process was repeated 100 times and the average values of performance indices were reported. For performance evaluation, we only evaluated the average accuracy defined as the total number of correctly predicted patients divided by the total number of patients. We ignored other evaluation metrics (e.g. precision, recall, area under the ROC curve, etc.) because the structure of the dataset was generally balanced, meaning the number of patients from each group was relatively similar to each other. The second task, which combines two PAH groups (HPAH plus IPAH) into one target group, provided a slightly unbalanced structure; but should not be considered as highly skewed.

In summary, for each target grouping and filtering method combination, we performed ANOVA-based gene ranking/selection, SIRRFE-based refinement, and three different machine learning algorithm-based training and prediction routines; namely, ANOVA+SIRRFE+LDA, ANOVA+SIRRFE+SVM, and ANOVA+SIRRFE+ANN. Additionally, we also run the experiments without using SIRRFE (ANOVA+LDA, ANOVA+SVM, and ANOVA+ANN) to verify its effectiveness.

## Supporting information

S1 Appendix Original array data. (ZIP)

S2 Appendix Original array data. (ZIP)

S3 Appendix IQR-based threshold detection report, codes and detailed modeling results files. (ZIP)

S1 Fig Detailed clustering of patient expression profiles based on conventional unsupervised hierarchical clustering indicated poor patient group separation compared to machine learning-based models. (PDF)
