# Supplementary material for: Machine learning-based microarray analyses indicate low-expression genes might collectively influence PAH disease
Source: PLoS Comput Biol. 2019 Aug 12;15(8):e1007264. doi: 10.1371/journal.pcbi.1007264 (PMC6705875; doi:10.1371/journal.pcbi.1007264)
Supplement: S3 Appendix — (ZIP) [file pcbi.1007264.s003.zip › code4all/IQR_Threshold_Detection.docx]

**Inter-quartile range (IQR) method for detecting the threshold probe intensity value of the PAH dataset**


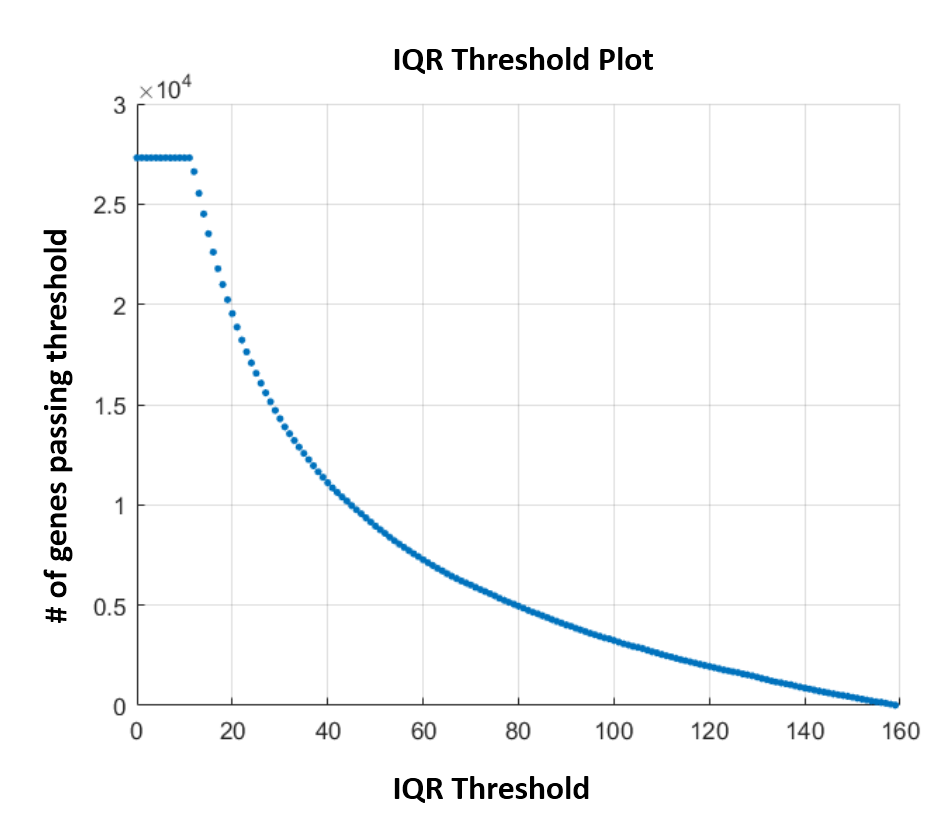


**Fig. S1 Plot of the inter-quartile range (IQR) profile of the whole PAH dataset.**


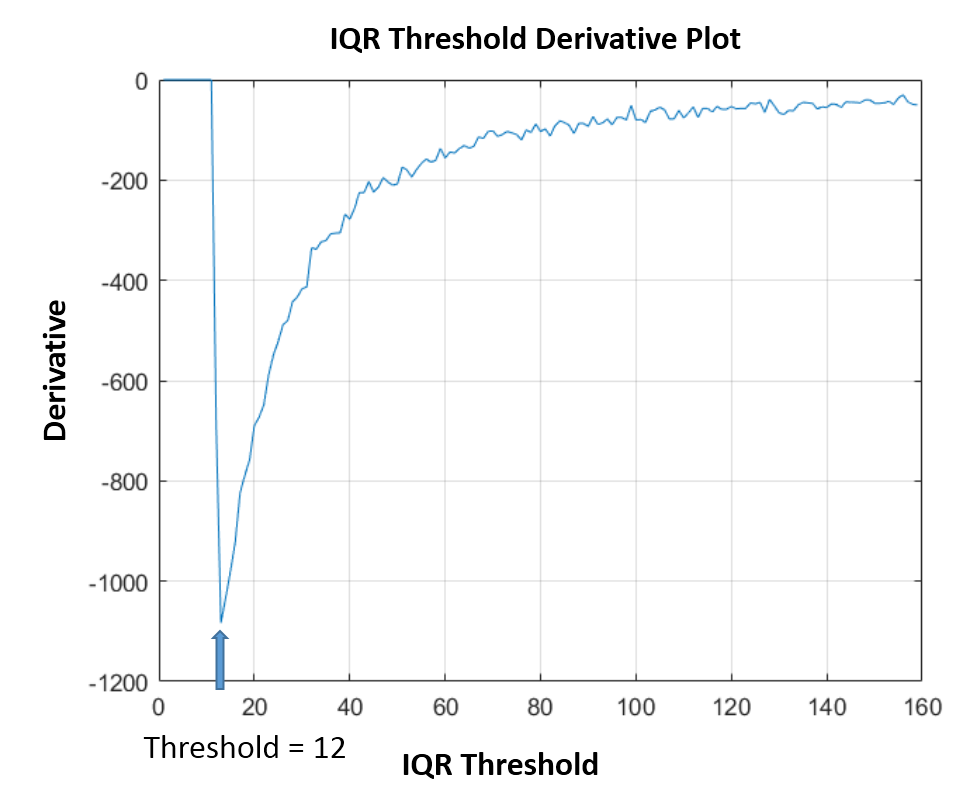


**Fig. S2 Plot of the first derivative of the inter-quartile range (IQR) profile of the whole PAH dataset, which identifies the optimal threshold probe intensity value of 12.**

The entire PAH dataset was analyzed using the IQR method [1-3]. The identified optimal threshold was 12 (probe intensity value after RMA normalization). Therefore, any gene with an average normalized probe intensity value smaller than 12 will be eliminated by IQR filtering method, commonly used by many bioinformatics tools such as the Bioconductor package. We kept the original probe intensity value in Fig. S1 instead of using the IQR value, because we are interested in finding the precise probe intensity cutoff value. The identified threshold value as indicated in Fig. S2 can be converted back to IQR value using the following equation:

**12/160 × 4 = 0.3**

1. Chockalingam,S. *et al*. (2016) Microarray data processing techniques for genome-scale network inference from large public repositories. Microarrays, 5, 23.
2. Kapetis,D. *et al.* (2012). AMDA 2.13: A major update for automated cross-platform microarray data analysis. BioTechniques 2012, 53, 33.
3. Aluru,M. *et al.* (2012) Reverse engineering and analysis of large genome-scale gene network. Nucleic Acids Res., 41, e24.
