# Supplementary material for: Machine learning-based microarray analyses indicate low-expression genes might collectively influence PAH disease
Source: PLoS Comput Biol. 2019 Aug 12;15(8):e1007264. doi: 10.1371/journal.pcbi.1007264 (PMC6705875; doi:10.1371/journal.pcbi.1007264)
Supplement: S3 Appendix — (ZIP) [file pcbi.1007264.s003.zip › code4all/PAH Report.pdf]

PAH Report:

Task 1: We constructed models that classify each patient into four different classes (groups), including: 1) Control, 2) Heritable PAH, 3) Mutations but no Disease, and 4) Sporadic/idiopathic PAH.

Data Prep Methods (# of genes used) / Classification Algorithms		All Data (54613)	All Genes>12 identified by IQR (385597)	All Group Ave>128 (14524)	All Group Ave>256 (10230)	At Least One Group Ave>128 (15794)	At Least One Group Ave<128 (40089)†	At Least One Group Ave>256 (11164)	At Least One Group Ave<256 (44383)
ANOVA +SIRRFE +LDA	Maximum Accuracy	0.9079	0.8605	0.7632	0.7642	0.7474	0.9795‡	0.7705	0.9653
	# of Genes Used	335	325	917	794	876	199‡	832	287
ANOVA +SIRRFE +SVM	Maximum Accuracy	0.9	0.8221	0.7347	0.7205	0.7674	0.9742‡	0.7463	0.9337
	# of Genes Used	58	41	182	8	218	31‡	19	104
ANOVA +SIRRFE +ANN	Maximum Accuracy	0.8679	0.7774	0.6879	0.6605	0.7132	0.94	0.68	0.9237
	# of Genes Used	32	41	191	221	43	87	22	74

Task 2: We constructed models that classify each patient into two different classes (groups), including: 1) Control, and 2) The Combination of Heritable PAH and Sporadic/idiopathic PAH.

Data Prep Methods (# of genes used) / Classification Algorithms		All Data (54613)	All Genes>12 identified by IQR (385597)	All Group Ave>128 (14524)	All Group Ave>256 (10230)	At Least One Group Ave>128 (15794)	At Least One Group Ave<128 (40089)†	At Least One Group Ave>256 (11164)	At Least One Group Ave<256 (44383)
ANOVA +SIRRFE +LDA	Maximum Accuracy	1	1	0.9938	0.9962	0.9992	1‡	0.9938	1
	# of Genes Used	13	16	12	188	419	8‡	18	18
ANOVA +SIRRFE +SVM	Maximum Accuracy	1	1	1	0.9946	0.9908	1‡	0.9985	1
	# of Genes Used	13	13	13	56	9	8‡	21	12
ANOVA +SIRRFE +ANN	Maximum Accuracy	1	1	1	0.9931	0.9938	1	0.9923	1
	# of Genes Used	24	14	13	55	62	16	19	27

Task 3: We constructed models that classify each patient into two different classes (groups), including: 1) Heritable PAH, and 2) Mutations but no Disease.

Data Prep Methods (# of genes used) / Classification Algorithms		All Data (54613)	All Genes>12 identified by IQR (385597)	All Group Ave>128 (14524)	All Group Ave>256 (10230)	At Least One Group Ave>128 (15794)	At Least One Group Ave<128 (40089)†	At Least One Group Ave>256 (11164)	At Least One Group Ave<256 (44383)
ANOVA +SIRRFE +LDA	Maximum Accuracy	1	1	0.954	0.943	0.946	1	0.984	1
	# of Genes Used	22	130	10	10	13	15	11	16
ANOVA +SIRRFE +SVM	Maximum Accuracy	1	1	0.941	0.923	0.979	1‡	0.971	1
	# of Genes Used	17	14	11	13	22	10‡	15	23
ANOVA +SIRRFE +ANN	Maximum Accuracy	1	1	0.941	0.924	0.961	1	0.954	1
	# of Genes Used	49	115	12	16	25	30	19	35

†Selecting genes with at least one group average < 128 appeared to be the optimal method.

‡Indicates the best-performing models identified within each task judging from the maximum accuracy (the greater the better) and number of genes used for achieving this accuracy (the less the better).

Underlined numbers/texts indicate externally linked files of error plots and ranked gene/probe names.
