# Supplementary material for: Machine learning-based microarray analyses indicate low-expression genes might collectively influence PAH disease
Source: PLoS Comput Biol. 2019 Aug 12;15(8):e1007264. doi: 10.1371/journal.pcbi.1007264 (PMC6705875; doi:10.1371/journal.pcbi.1007264)
Supplement: S1 Fig — (PDF) [file pcbi.1007264.s004.pdf]

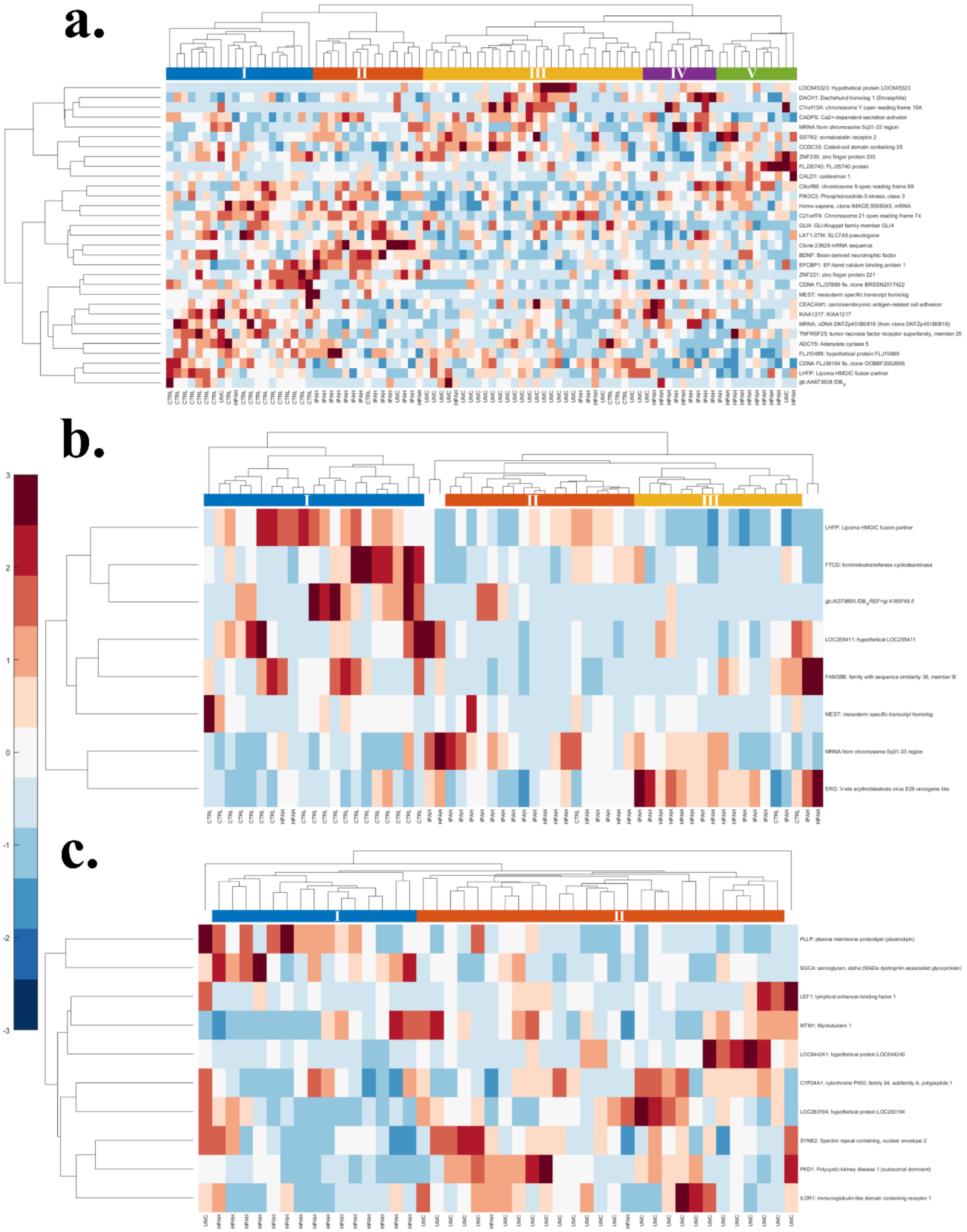

Fig S1. Detailed clustering of patient expression profiles based on conventional unsupervised hierarchical clustering indicated poor patient group separation compared to machine learning-based models. Clustered heat maps were generated using (a) the

identical 31 ranked genes used for constructing the support vector machine-based (SVM) multiclass classification model, which distinguishes each individual PAH patient group (Control *vs.* IPAH *vs.* HPAH *vs.* UMC) with an accuracy value of 0.97; (b) the identical eight ranked genes used for constructing SVM-based classification model, which distinguish the healthy control *vs*. the combination of HPAH and IPAH with a perfect accuracy; and (c) the identical 10 genes used for constructing the SVM-based classification model, which separates HPAH *vs*. UMC with a perfect accuracy All expression levels are standardized along the row dimension and the hierarchical cluster trees are generated using unsupervised linkage functions based on the inner squared/unweighted average distance between all pairs of objects in any two clusters. Clustering of patients are depicted by the dendrogram on top with cluster numbers labeled in Latin numbers. Higher expression levels are represented as increasing red, lower ones are represented as increasing blue, and white represents close to the average. All gene names are appended to the right of the map with clustering dendrogram appended to the left.
